# Bv8 mediates myeloid cell migration and enhances malignancy of colorectal cancer

**DOI:** 10.3389/fimmu.2023.1158045

**Published:** 2023-04-05

**Authors:** Xiaomeng Li, Enqiang Chang, Jiang Cui, Hailin Zhao, Cong Hu, Kieran P. O’Dea, Nikhil Tirlapur, Gianfranco Balboni, Jiaqiang Zhang, Liming Ying, Daqing Ma

**Affiliations:** ^1^ Division of Anaesthetics, Pain Medicine and Intensive Care, Department of Surgery and Cancer, Faculty of Medicine, Imperial College London, Chelsea and Westminster Hospital, London, United Kingdom; ^2^ Department of Anaesthesiology and Perioperative Medicine, Henan Provincial People’s Hospital, People’s Hospital of Zhengzhou University, Zhengzhou, China; ^3^ Division of Translational Critical Care, Department of Surgery and Cancer, Faculty of Medicine, Imperial College London, Chelsea and Westminster Hospital, London, United Kingdom; ^4^ Department of Life and Environmental Sciences, University of Cagliari, Monserrato, Italy; ^5^ National Heart and Lung Institute, Imperial College London, Molecular Sciences Research Hub, London, United Kingdom

**Keywords:** colorectal cancer, prokinectin 2, Bv8, myeloid cells, malignancy

## Abstract

Colorectal cancer (CRC) is the third most predominant malignancy in the world. Although the importance of immune system in cancer development has been well established, the underlying mechanisms remain to be investigated further. Here we studied a novel protein prokineticin 2 (Prok2, also known as Bv8) as a key pro-tumoral factor in CRC progression in *in vitro* and *ex vivo* settings. Human colorectal tumor tissues, myeloid cell lines (U937 cells and HL60 cells) and colorectal cancer cell line (Caco-2 cells) were used for various studies. Myeloid cell infiltration (especially neutrophils) and Bv8 accumulation were detected in human colorectal tumor tissue with immunostaining. The chemotactic effects of Bv8 on myeloid cells were presented in the transwell assay and chemotaxis assy. Cultured CRC cells treated with myeloid cells or Bv8 produced reactive oxygen species (ROS) and vascular endothelial growth factor (VEGF). Furthermore, ROS and VEGF acted as pro-angiogenesis buffer in myeloid cell-infiltrated CRC microenvironment. Moreover, myeloid cells or Bv8 enhanced energy consumption of glycolysis ATP and mitochondria ATP of CRC cells. Interestingly, myeloid cells increased CRC cell viability, but CRC cells decreased the viability of myeloid cells. ERK signalling pathway in CRC cells was activated in the presence of Bv8 or co-cultured myeloid cells. In conclusion, our data indicated the vital roles of Bv8 in myeloid cell infiltration and CRC development, suggesting that Bv8 may be a potential therapeutic target for colorectal cancer-related immunotherapy.

## Introduction

Colorectal cancer (CRC), also known as colorectal adenocarcinoma, is the third most predominant malignancy following lung and breast cancer in the world (1). Survival rate of CRC patients varies among different countries and regions, largely dependent on economical income and access to early screening and treatment (2). Clinicopathological data suggested that the combination of immunotherapy and local surgery, radiotherapy or chemotherapy remarkably enhances CRC patients’ tumor-free survival (3). Despite that combination therapy of immunotherapy and other therapeutic approaches are highly recommended for CRC due to its great promise and fewer side effects, challenges such as drug resistance still exist (4). Therefore, there is a continuous urge to investigate the nature of immunity in CRC environment and find potential therapeutic target (5).

The immune system plays a “double edged saw” role in CRC of both suppressing its initiation and promoting its angiogenesis and metastasis (6). Numerous immune cells are featured with both anti-tumorigenic and pro-tumorigenic responses *via* the release of cytokines and chemokines (7). Recent studies revealed that Bv8, also known as prokinectin 2 (Prok2), regulates myeloid-cell-dependent tumor angiogenesis (8). Higher plasma level of Bv8 in CRC patients than in healthy volunteers has been noticed (9). Previous *in vitro* and *in vivo* study showed that anti-Bv8 treatment reduced the kinetics of tumor-associated CD11b^+^Gr1^+^ cells, indicating the regulatory role of Bv8 in myeloid cell mobilization (8). These studies hinted the possibility of accumulation of Bv8 in CRC sites with chemokine-like features. The contribution of Bv8 in the development CRC has also been reported. For example, Kurebayashi et al. demonstrated that Bv8 gene transfection enhanced angiogenesis and tumor growth of CRC in mice model (10). Yoshida et al. also reported that Bv8 overexpression may be a prognostic indicator of the CRC stage, especially for patient with lymphatic invasion or lymph node metastasis (11). Moreover, the reciprocal relationship between Bv8 and ROS has recently been reported. Cheng et al. demonstrated that Bv8 mRNA increased after ROS treatment in both *in vitro* and *in vivo* settings (12). On the other hand, Bv8 offered neuroprotection by reducing the cytotoxic level of ROS in astrocytes cultures (13). Another *in vitro* study also revealed that exogenous Bv8 reduced lipid ROS generation in primary neuronal cells (14). Although similar studies in cancers are limited, it is reasonable to hypothesize that Bv8 might have a similar protective effect on cancer cells *via* ROS regulation. Since ROS is one of the most established by-products during mitochondrial energy generation, namely, ROS-induced ROS release (RIRR) (15), we also hypothesize that Bv8 may regulate mitochondrial metabolism at the same time as ROS.

So far, there is still a gap of the knowledge of Bv8-induced malignancy in CRC at cellular level. It is therefore worth conducting a comprehensive functional study of Bv8 on CRC cells and tissues. Herein we report our investigation of the impact of Bv8 on myeloid cell infiltration and its malignant effects on CRC cells. We detected myeloid cell infiltration and Bv8 accumulation in human CRC tissue, which might be associated with Bv8-induced chemotactic effects on myeloid cells. We also found that myeloid cell or Bv8 enhanced the production of ROS, VEGF and ATP in CRC cells, which might act as pro-tumoral buffer in myeloid cell-infiltrated CRC microenvironment. Furthermore, our data suggested that ERK signalling pathway might contribute to these Bv8-mediated effects.

## Materials and methods

### Cell lines and co-cultures

Human colorectal adenocarcinoma (Caco-2) cells (ECACC, Wiltshire, UK) were cultured in Gibco Dulbecco’s Modified Eagle Medium (DMEM, ThermoFisher SCIENTIFIC, Paisley, UK) supplied with 10% heat-inactivated foetal bovine serum (FBS) and 1% penicillin-streptomycin (ThermoFisher SCIENTIFIC). Both human myeloid cell lines HL60 (ECACC, Wiltshire, UK) (mimicking neutrophil response) and U937 cells (ATCC, Virginia, USA) (mimicking monocyte response) were cultured in Gibco RPMI media 1640 (ThermoFisher SCIENTIFIC) supplemented with 10% FBS and 1% penicillin-streptomycin. All cell lines were incubated at 37°C with 5% CO_2_ supply and culture medium was changed every 2 days. Caco-2 cell passaging was done when cells were 90%-100% confluent with 0.05% trypsin with 0.6 mM ethylenediaminetetraacetic acid (EDTA) (ThermoFisher SCIENTIFIC),. During 24-hour co-cultures, about 10^5^ HL60 or U937 cells were added into 24-well plate containing approximately 2.4 x10^5^ Caco-2 cells in RPMI or DMEM medium, respectively. Suspension cells (HL60 and U937 cells) and adherent cells (Caco-2 cells) were collected separately after co-cultures for further analysis.

### Human blood and cancer *ex vivo* samples

After ethic approval was obtained from NRES Committee London - Camden and Islington (reference 15/LO/0933), whole human blood was kindly given by our colleagues. Neutrophils were isolated by density gradient separation method using 3% Dextran T500 (Sigma-Aldrich, Dorset, UK). The purity and cell count of neutrophils were assessed by flow cytometry analysis using anti-CD45 and anti-CD66b antibodies (Biolegend, San Diego, CA) and counting beads (Sigma-Aldrich) respectively. Primary tumor tissues and normal tissues from cancer patients were kindly given by our colleagues from Henan Provincial Hospital, Zhengzhou, Henan, China (Ethics approval no. 201944). To minimize individual variation, all tumor tissue and its NAT were harvested from the same patient and analyzed for comparison. All tissue specimens were formalin-fixed and paraffin-embedded when received. Specimens were stored at -20°C for future use.

### Transwell assay

Approximately 10^5^ U937 cells were suspended in RPMI containing 10% FBS and placed in the upper chamber with 8μm pore size. RPMI alone or RPMI with chemoattractants was placed into the lower chamber. Cells were then incubated at 37°C with 5% CO_2_ for 24 hours. After which, migrated cells in the lower chamber were counted by flow cytometry analyses using counting beads.

### Chemotaxis assay

CellDirector 2D (Gradientech, Uppsala, Sweden) was used to assess the chemotactic effect of Bv8 on human neutrophils (16). Neutrophils were freshly collected and injected into poly-l-lysine-coated (Sigma-Aldrich) CellDirector 2D. After cells were adherent to the chamber surface, 1 ml of 100 nM recombinant human Bv8 (Abcam, Cambridge, UK) in RPMI and 1 ml of RPMI were pumped into the chamber at 1 µl/min *via* two syringes (17). This created a continuous concentration gradient of Bv8 in the chamber. Bright-field video was captured for 2 hours at a rate of 0.5 fpm using a Nikon TE2000 inverted optical microscope equipped with a 10× objective and a Cool-View EM1000 EMCCD camera (Photonic Sciences, UK).

### Western blotting

Cells were lysed using lysis buffer and centrifuged at 10000 r.p.m. for 30 minutes at 4°C. The supernatant was collected, and the total protein concentration was quantified using Bradford protein assay (Bio-Rad Laboratories, Hercules, CA, USA). The protein extracts were heated for 10 min at 95°C and NuPAGE 4-12% Bis-tris gel (Invitrogen) was used to load protein samples for electrophoresis. After the electrophoresis, protein membranes were incubated with anti-phospho-p44/42 MAPK (Erk1/2), anti-p44/42 MAPK (ErK1/2)), anti-AKT antibody, anti-phospho-AKT, anti-STAT3 antibody, anti-phospho-STAT3 antibody (all from Cell Signalling, Massachusetts, USA) or anti-GAPDH antibody (Sigma-Aldrich, Missouri, USA) overnight at 4°C. The membrane was then incubated with anti-rabbit IgG, HRP linked antibody (Cell signalling) for 1hr. The membrane was visualized through enhanced chemiluminescence system (Santa Cruz). Protein bands were captured by Syngene GeneSnap software (Syngene, Cambridge, UK) and analyzed by Image J (NIH, Bethesda, USA).

### Immunofluorescent staining

Approximately 50,000 Caco-2 cells were seeded on coverslips placing in the 24-well plate and allowed to grow overnight. After 1-100nM Bv8 treatment or co-cultures, Caco-2 cells were incubated with anti-Bv8 antibodies (Abcam, Cambridge, UK), anti-VEGF antibodies (Abcam) or myeloperoxidase (MPO) antibodies (Santa Cruz, Texas, US) at 4°C overnight. After cells were incubated with secondary antibodies, mounting medium DAPI (Vector laboratories, inc, Burlingame, USA) was used to mount the cells. Fluorescent images were captured using wide-field fluorescent microscopy and fluorescent intensity was evaluated by Image J. In order to investigate the effect of Bv8 and Erk signalling pathway on VEGF expression in CRC cells, Bv8, Bv8 receptor antagonist PKRA7 (Merk, New Jersey, US), another Bv8 receptor antagonist PC-1 (kindly donated by Professor Gianfranco Balboni, Department of Life and Environmental Sciences, University of Cagliari, Italy) and Erk1/2 inhibitor SCH772984 (SCH, Thermo Fisher Scientific, USA) were added to CRC cell line.

### Flow cytometry

Cells were stained with Dihydrorhodamine 123 (DHE) (Thermo Fisher Scientific), a non-fluorescent indicator that binds to ROS, at 37°C in dark for 5 minutes. For each sample, approximately 50000 cells were analyzed with a flow cytometer (CyAn ADP, Beckman Coulter, US). Mean fluorescent intensity of ROS and relative histogram was analyzed and generated by FlowJo (Becton, Dickinson & Company, USA)

### Cell viability assay

Caco-2 cells in each well of a 96-well plate were co-cultured with HL60 cells, U937 cells or cell medium (extracted from HL60 or U937 cell cultures). Another cohort Caco-2 cultures were treated with 1 nM Bv8, 1 μM of PC-1 + 1 nM Bv8, 1 μg/ml of PKRA7 + 1 nM Bv8, and DMSO (vehicle control). Since Caco-2 cells are adherent cells and HL60 and U937 cells are suspension cells, Caco-2 cells and HL60 or U937 cells were collected separately from the co-culture setting after 24 hours. CCK-8 assay was then carried out on Caco-2 cells, HL60 cells and U937 cells to determine their viability with cell counting kit-8 (CCK-8) (Sigma-Aldrich) applied to each well for 4 hours at 37°C. Optical density (OD) value of each sample was then measured by a microplate reader at 450 nm wavelength. Cell viability was calculated as [OD (treatment) – OD (blank)]/[OD (control) – OD (blank)]X 100%.

### Real-time ATP assay

One day before Seahorse XF Real-Time ATP Rate Assay (Agilent, California, USA), approximately 10^4^ Caco-2 cells accompanied with various treatment were seeded in each well of the Seahorse XF Microplate (Agilent). The sensor cartridge (Agilent) was hydrated at 37°C with no CO_2_ supply. Assay media was prepared using 100 ml of Seahorse XF DMEM medium, 10 mM of XF glucose, 1 mM of XF Pyruvate and 2 mM of XF glutamine (Agilent). Cells were washed with warmed assay medium and incubated at 37°C with no CO_2_ supply for 60 minutes prior to the assay. 1.5 μM of oligomycin and 0.5 μM Rotenone + Antimycin A was loaded into the sensor cartridge that were injected into cell medium at the 20^th^ minute and the 40^th^ minute respectively during the assay. The serial addition of oligomycin and Rotenone + Antimycin during the assay inhibited mitochondrial ATP production and electron transportation, respectively, facilitating the quantification of ATP production rate. Data was analyzed using Seahorse XF Real-Time ATP Rate Assay Report Generator (Agilent).

### Statistical analysis

Numerical data were presented as mean ± SD or median (range) together with dot plot where appropriate. Anderson-Darling, D’Agostino-Pearson omnibus, Shapiro-Wilk, and Kolmogorov-Smirnov test were used to determine data distribution using GraphPad Prism (GraphPad Software, La Jolla, CA, USA). For normally distributed data of two groups only, student’s t test was used for comparison; Otherwise Mann-Whitney U test was used for non-parametric data. For normally distributed data of more than two groups, data were analyzed by one or two- way ANOVA followed by Student-Newman-Keuls test for multi comparisons; Otherwise, Kruskal-Wallis test followed by Dunn’s test was used for non-parametric data. A p-value less than 0.05 was considered to be of statistical significance.

## Results

### Bv8 expression increases in malignant tumors and co-localizes with CRC-infiltrated myeloid cells

**Figure 1:**
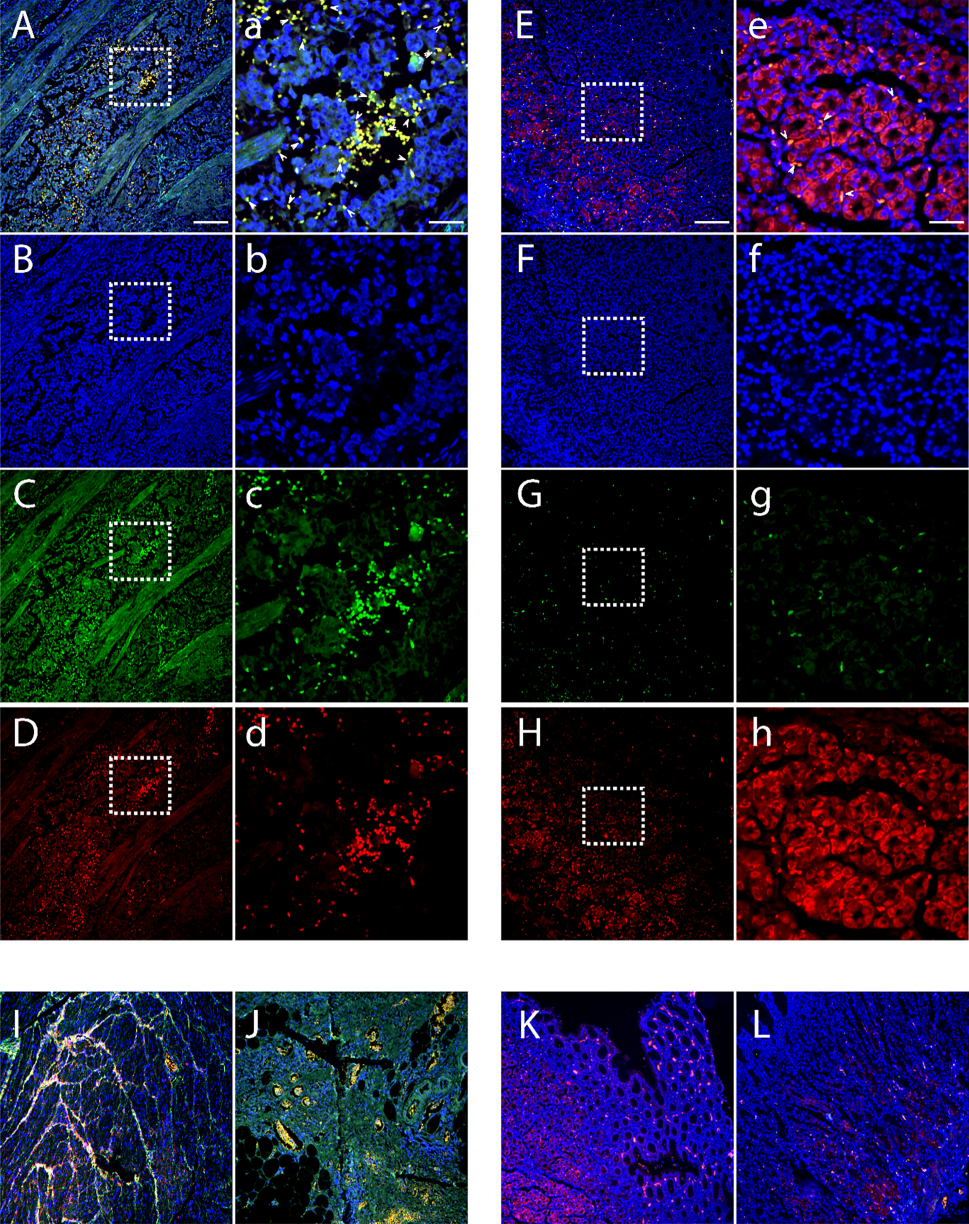
Location of CRC-infiltrated myeloid cells and Bv8 in tumor tissue and NAT of 3 patients with CRC. **(A–D)** Tumor tissue of a CRC patient. Sample was labelled with DAPI (blue), MPO (red) and anti-Bv8 (green) through immunofluorescent staining. MPO was used as myeloid cell marker. Arrowheads in insets depict co-localization of Bv8 and infiltrated myeloid cells. **(E–H)** NAT of the same CRC patient were labelled with DAPI (blue), MPO (red) and anti-Bv8 (green) through immunofluorescent staining. MPO was used as myeloid cell marker. Arrowheads in insets depict co-localization of Bv8 and infiltrated myeloid cells. **(I–L)** The expression and location of myeloid cells and Bv8 in another two CRC patients. Small letters a-h indicate the zoom-in image of the squared area from A-H respecively. Scale bar: 200 µm **(A–L)**, 50 μm **(A–H)**.

We compared Bv8 expression from tumor tissue (collected from primary tumor site) and normal tissue adjacent to the tumor (NAT) from patients with CRC through immunofluorescence staining. Tissues from patients with prostate or breast cancer were also included for comparison. As presented in **Supplemental Figure 1**, malignant tumor microenvironment led to a significant increase of Bv8 expression in all cancer types. To further determine the association between Bv8 and CRC-infiltrated myeloid cells, we performed additional staining of myeloid cells (mostly neutrophils and monocytes) in CRC tissue using MPO antibody. In accordance with **Supplemental Figure 1**, higher Bv8 expression was detected in the CRC tumor tissue than its NAT (**Figure 1**). Furthermore, zoom-in images (**Figures 1A–D**) indicates co-localization of myeloid cells and Bv8 proteins in CRC tumor tissue, suggesting that tumor-infiltrated myeloid cells may be a potential source of Bv8 in CRC.

### Bv8 induces the chemotaxis of myeloid cell

**Figure 2:**
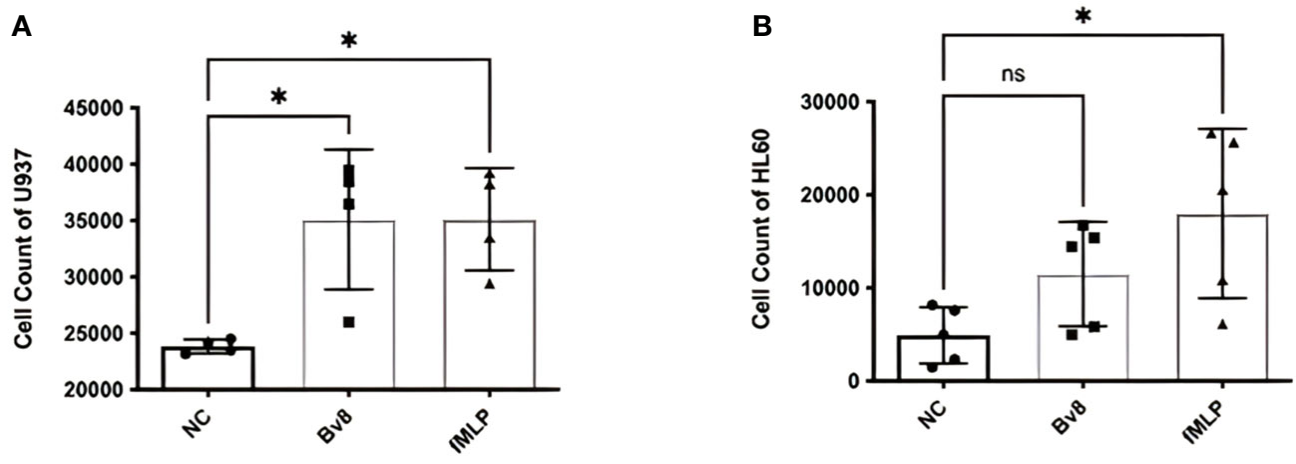
Chemotactic effects of Bv8 on myeloid cell lines. **(A)** The number of recruited U937 (n=4) cells moving towards Bv8 (10 µM) or fMLP (10 µM) was assessed by transwell migration assay. **(B)** The number of recruited HL60 cells (n=5) cells moving towards Bv8 or fMLP. All data were presented as mean ± SD. Mann-Whitney U test followed by Dunn’s test was used for statistical analysis. *p<0.05, ns, not significant. n refers to the number of independent experiments.

*In vitro* migration study using myeloid cell lines HL60 and U937 showed that significantly increased number of U937 cells migrated towards the lower chamber, where Bv8 or fMLP (a well-known potent chemoattractant for myeloid cells) was added (**Figure 2A**). There was an increase of HL60 cells towards lower chamber filled with Bv8 compared to the naïve controls but did not reach to a level of statistical significance (**Figure 2B**). To further study the chemotactic pattern of primary myeloid cells, a real-time chemotaxis assay of human neutrophils in response to Bv8 concentration gradient (0 nM to 100 nM) was carried out (**Supplemental Figure 2A**). The location of individual neutrophils with movement traces were presented at time points of 0 minute and the 40th minute, respectively (**Supplemental Figure 2B**). Among all 216 neutrophils being tracked, 76% of the cells moved towards the direction of lower Bv8 concentration (right towards 0nM of Bv8, P=3.5E-20) (**Supplemental Figure 2B**). Real-time video was uploaded as **Supplementary File 2**.

### Bv8 induces Erk activation in the CRC cells

**Figure 3:**
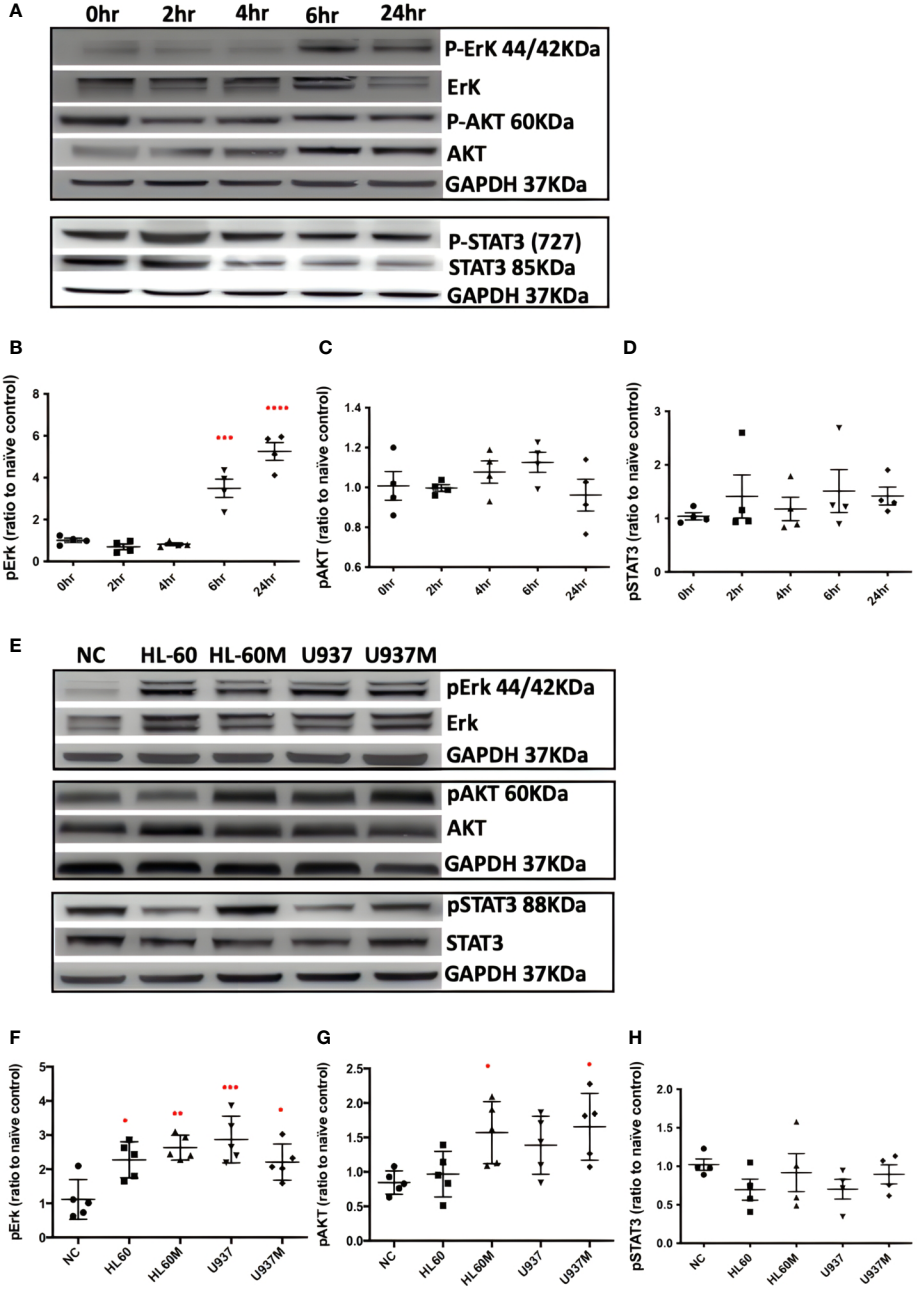
Both Bv8 and myeloid cells activated Erk signalling pathway in Caco-2 cells. **(A)** Caco-2 cells were treated with 1nM of Bv8 for various of time period. Western blot was performed and phosphorylation of Erk, AKT and STAT3 in Caco-2 cells were shown as densitometry. **(B)** Protein density of phosphorylated Erk (n=5) after Bv8 treatment. **(C)** Protein density of phosphorylated AKT (n=5) after Bv8 treatment. **(D)** Protein density of phosphorylated STAT3 (n=4) after Bv8 treatment. **(E)** In order to investigate if myeloid cell infiltration also induced activation of potential signalling pathways, HL60 cells, U937 Cells and cell medium extracted from both cell cultures were given to Caco-2 cells for 24 hours. Phosphorylation of Erk, AKT and STAT3 in Caco-2 cells were shown as densitometry. **(F)** Protein density of phosphorylated Erk (n=5) after co-culture treatment. **(G)** Protein density of phosphorylated AKT (n=5) after co-culture treatment. **(H)** Protein density of phosphorylated STAT3 (n=4) after co-culture treatment. Protein density was analyzed as ratio to naïve control. All statistic data was illustrated as mean ± SD. Student’s t test was used for statistical analysis. *p<0.05, **p<0.01, ***p<0.001, ****p<0.0001. NC, naïve control; HL60, co-cultured with HL60 cells; HL60M, co-cultured with HL60 medium; U937, co-cultured with U937 cells; U937M, co-cultured with U937M. n refers to the number of independent experiments.

After treating with 1 nM of Bv8 for 2 to 24 hours, Caco-2 cells were collected and the phosphorylated Erk, phosphorylated AKT and phosphorylated STAT3 were determined through western blotting. We noticed that pErk in Caco-2 was activated after 6 to 24-hour treatment of Bv8 (**Figures 3A, B**). No significant change of pAKT or pSTAT3 in Caco-2 cells was shown after Bv8 treatment (**Figures 3C, D**). Since myeloid cells are recruited by Bv8 to the tumor microenvironment of CRC, the co-culture of myeloid cells and CRC cells was set up to mimic myeloid cell infiltration and to further explore the role of CRC-infiltrated myeloid cells in activation of these signalling pathways. Co-cultures and their extracted culture media significantly increased the expression of pErk in Caco-2 cells (**Figures 3E, F**). The extracted culture medium from HL60 cells or U937 cells also enhanced pAKT expression in Caco-2 cells (**Figure 3G**). No significant change of pSTAT3 in Caco-2 cells was shown after co-culture treatment (**Figure 3H**).

### Bv8 affects the malignancy of CRC cells

**Figure 4:**
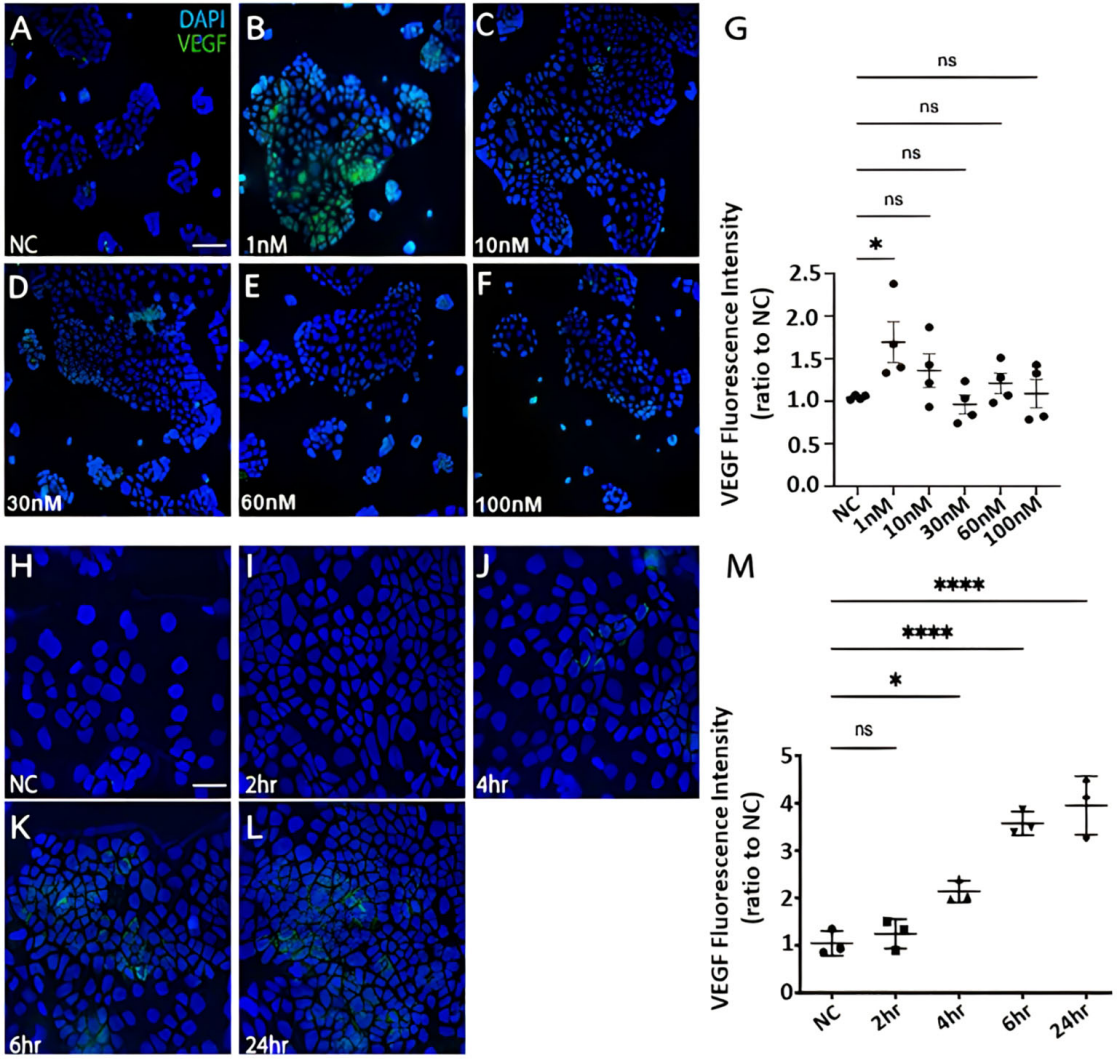
The effect of Bv8 on VEGF expression in CRC cells. **(A)** The immunostaining fluorescence data presented VEGF expression (green) in Caco-2 cells that were treated with 0 nM Bv8 (naïve control). **(B)** VEGF expression in Caco-2 cells that were treated with 1 nM Bv8. **(C)** VEGF expression in Caco-2 cells that were treated with 10 nM Bv8. **(D)** VEGF expression in Caco-2 cells that were treated with 30 nM Bv8. **(E)** VEGF expression in Caco-2 cells that were treated with 60nM Bv8. **(F)** VEGF expression in Caco-2 cells that were treated with 100 nM Bv8. **(G)** Data were then analyzed as ratio to the fluorescence intensity of naïve control (n=4). 1 nM was the only concentration of Bv8 that caused significant increase of VEGF expression in Caco-2 cells after 24-hour treatment. **(H)** VEGF expression in Caco-2 cells treated with 1 nM Bv8 for 0 hour (naïve control). **(I)** VEGF expression in Caco-2 cells treated with 1 nM Bv8 for 2 hours. **(J)** VEGF expression in Caco-2 cells treated with 1nM Bv8 for 4 hours. **(K)** VEGF expression in Caco-2 cells treated with 1 nM Bv8 for 6 hours. **(L)** VEGF expression in Caco-2 cells treated with 1 nM Bv8 for 24 hours. **(M)** Data were then analyzed as ratio to the fluorescence intensity of naïve control (n=3). The data indicated that Bv8 started to induce extensive production of VEGF in Caco-2 cells after 4 hours. All data were presented as mean ± SD. Student’s t test was used for statistical analysis. *p<0.05, ****p<0.0001, ns, not significant. NC, naïve control. Scale bar: 100 μm for **(A-F)**, 50 μm for **(H-L)**. n refers to the number of independent experiments.

**Figure 5:**
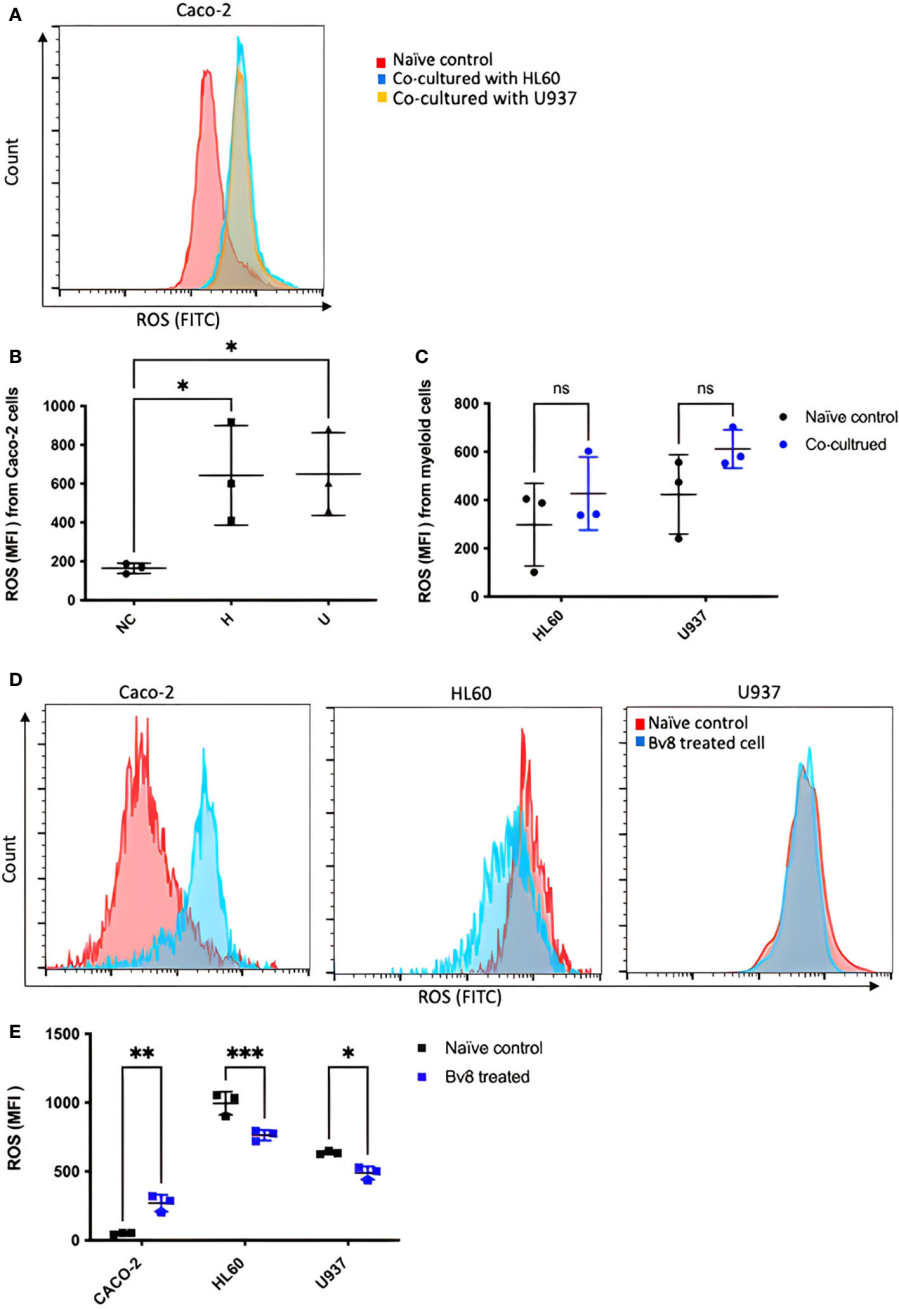
ROS production from CRC cells and myeloid cells after co-culture or Bv8 treatment. ROS production from both CRC cells and myeloid cells after 24-hour co-culture was assessed by flow cytometry. **(A)** The histogram of fluorescent intensity of ROS from Caco-2 cells, where the peak of treatment curve shifted to the right (blue: Caco-2 cells co-cultured with HL60, yellow: Caco-2 cells co-cultured with U937) compared to naïve control (red). **(B)** Co-culturing with HL60 cells and U937 cells significantly enhanced ROS production from Caco-2 cells (n=3). **(C)** No significant change of ROS production from either HL60 cells or U937 cells was observed after co-culturing with Caco-2 cells (n=3). **(D)** Histograms showed right-shift of ROS fluorescent intensity in Caco-2 cells and left-shift in HL60 cells and U937 cells (red: naïve control, blue: Bv8 treated cells). **(E)** Increased ROS in Caco-2 cells and decreased ROS level in HL60 cells and U937 cells after treated with 1 nM Bv8 for 24 hours (n=3). All data were presented as mean ± SD. Mann-Whitney U test followed by Dunn’s test was used for statistical analysis. *p<0.05, **p<0.01, ***p<0.001, ns, not significant, NC, naïve control; H, Caco-2 cells that were co-cultured with HL60 cells; U, Caco-2 cells that were co-cultured with U937 cells. n refers to the number of independent experiments.

CRC cells were incubated with various concentration of Bv8 from 1 nM to 100 nM for 24 hours (**Figures 4A–F**). Unexpectedly, only the lowest concentration of Bv8 (1 nM) triggered a significantly increased expression of angiogenic factor VEGF in CRC cells (**Figure 4G**). Furthermore, the expression of 1 nM Bv8-induced VEGF rose significantly from as early as 4 hours, up until 24 hours (**Figures 4H–M**), indicating that incubation time might not be a key factor in Bv8-induced VEGF expression in CRC cells. Moreover, addition of Erk inhibitor SCH significantly suppressed Bv8-induced VEGF expression (**Supplemental Figure 3**). It is possible that Erk signalling pathway may be involved in Bv8-induced VEGF expression in CRC cells. Since myeloid cells are recruited by Bv8, we also co-cultured CRC cells with myeloid cell lines. Our data suggested that only direct cellular interaction between CRC cells and HL60 cells or U937 cells, but not their medium, induced VEGF expression in CRC cells (**Supplemental Figure 4**). This indicated the important role of cell-cell interaction between myeloid cells and CRC cells in VEGF production in CRC cells. Similarly, direct cellular interaction of myeloid cells and CRC cells significantly increased the ROS production from Caco-2 cells (**Figures 5A, B**). Similar increase of ROS production from myeloid cells was observed but not reach to significant level (**Figure 5C**). Interestingly, Bv8 triggered more ROS production from Caco-2 cells but inhibited the ROS production from both myeloid cell lines (**Figures 5D, E**).

We found that cell-cell interaction suppressed myeloid cell viability but increased CRC cell viability. The cell viability of U937 was dramatically suppressed after co-culture with Caco-2 cells (**Supplemental Figure 5A**). On the other hand, Caco-2 cells presented an increased cell viability after co-cultured with U937 cells (**Supplemental Figure 5B**). Similar trend was seen after co-culture of HL60 cells and Caco-2 cells but no significance was reached. To further investigate if cell viability enhancement of Caco-2 was associated with myeloid cell-derived Bv8, Caco-2 cells were treated with Bv8 and Bv8 receptor antagonists. No significant change was noticed apart from Bv8 +PKRA7 treatment (**Supplemental Figure 5C**). SCH (Erk inhibitor) significantly decreased Bv8-enhanced cell viability of Caco-2 cells, suggesting that Erk signalling pathway might be involved in Bv8-induced cellular changes. No significant change of the expression of Bv8 receptor (PKR2) was found after co-culture treatment (**Supplemental Figure 6**).

**Figure 6:**
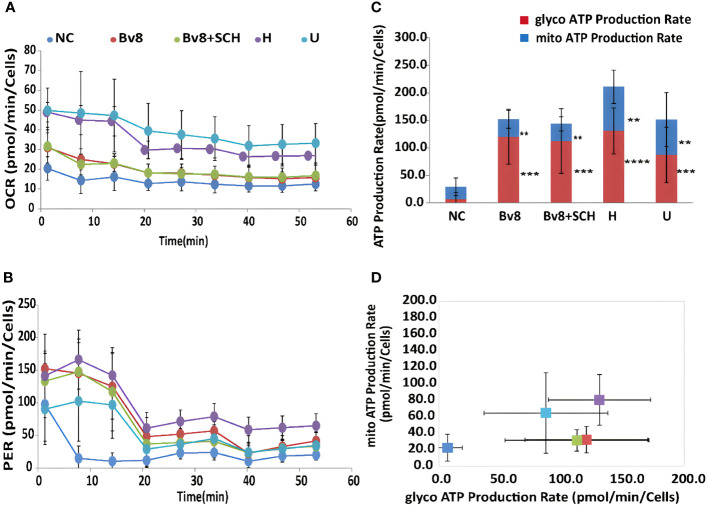
Bv8 or co-culturing with myeloid cells increased energy consumption of Caco-2 cells. **(A)** Real-time ATP production rate during mitochondrial respiration for 60 minutes and data was presented as oxygen consumption rate (OCR, pmol/min/cells) of Caco-2 cells. Caco-2 cells were treated with 1 nM of Bv8 (red), 500 nM SCH + 1 nM Bv8 (green), Hl60 cell (purple) and U937 cells (light blue) prior to ATP assay. **(B)** Real-time ATP production rate during glycolysis for 60 minutes and data was presented as proton efflux rate (PER, pmol/min/cells) of Caco-2 cells. **(C, D)** Both glycolytic ATP production rate and mitochondrial ATP production rate of Caco-2 cells were significantly increased after all treatment. Caco-2 cells were treated with 1 nM of Bv8 (red), 500 nM SCH + 1 nM Bv8 (green), Hl60 cell (purple) and U937 cells (light blue) prior to ATP assay. All data were presented as mean ± SD. Two-way ANOVA was used for statistical analysis followed by Student-Newman-Keuls test **p<0.01, ***p<0.001, ****p<0.0001. NC, naïve control; H, co-cultured with HL60 cells; U, co-cultured with U937 cells. n refers to the number of independent experiments.

Sixty-minute real-time ATP production from mitochondria (oxygen consumption rate, OCR) and glycolysis (proton efflux rate, PER) in Caco-2 cells were readily detected. Significantly increased mitochondrial (**Figure 6A**) and glycolysis (**Figure 6B**) ATP production rate of Caco-2 cells was found after they were treated with Bv8 (red) or myeloid cells (purple and light blue). ATP production inhibitors oligomycin and rotenone/antimycin A were injected at the 20th and the 40th minute respectively, where sharp drops were seen (**Figures 6A, B**). The mean glycolytic ATP production rate of naïve control Caco-2 cells, Caco-2 cells treated Bv8, Caco-2 cells treated with Bv8+SCH, Caco-2 cells co-cultured with HL60 cells and Caco-2 cells co-cultured with U937 cells were 7.0 ± 12.2, 120.3 ± 50.0, 112.6 ± 58.7, 131.0 ± 41.7 and 87.3 ± 50.4 pmol/min/cells respectively (**Figure 6D**). Significant increase of glycolytic ATP production rate was noticed between naïve control and all other treatment groups (**Figure 6C**). Moreover, mean mitochondrial ATP production rate of naïve control Caco-2 cells, Caco-2 cells treated Bv8, Caco-2 cells treated with Bv8+SCH, CAco-2 cells co-cultured with HL60 cells and Caco-2 cells co-cultured with U937 cells were 22.1 ± 16.2, 31.7 ± 16.1, 31.0 ± 13, 80.1 ± 30.6 and 64.2 ± 48.6 pmol/min/cells respectively (**Figure 6D**). Similar significance of mitochondrial ATP production rate was also found between naïve control group and all other treatment groups (**Figure 6C**).

## Discussion

**Figure 7:**
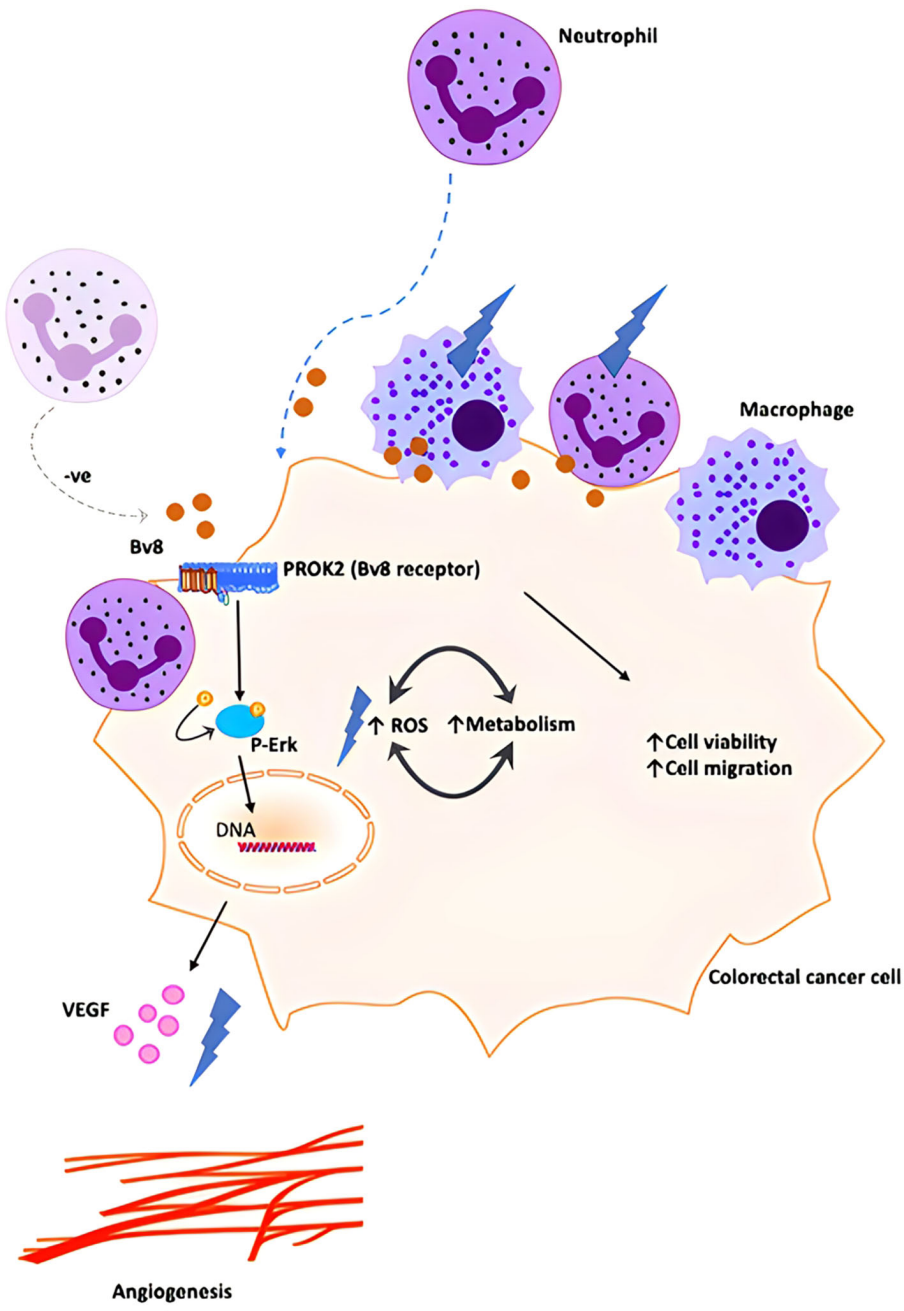
Schematic illustration of proposed orchestration of myeloid cells and CRC progression. We propose that small number of myeloid cells infiltrate into inflamed colorectal tissue and secrete low level of Bv8 at the pre-cancer stage of CRC. Bv8 act as chemoattractant for myeloid cells. However, it is possible that the myeloid cell-CRC cell system actively regulates Bv8 at a low level to establish a pro-tumoral microenvironment (for example, myeloid cells might contribute to the negative regulation of Bv8 production in Caco-2 cells, maintaining Bv8 at a relatively low level.). Bv8 also promote tumorigenesis of CRC by enhancing VEGF-mediated angiogenesis and cancer cell metabolism, potentially through ErK signalling pathway. With the migration of myeloid cells, myeloid cell infiltration occurred and Bv8 was accumulated in CRC tumor.

Our data demonstrated a higher expression of Bv8 in tumor tissue compared to NAT from the same *ex vivo* samples of patients with CRC. Co-localization of Bv8 and infiltrated myeloid cells in CRC tumor was also identified. Bv8 is likely to be a chemoattractant for myeloid cells. Bv8 and infiltrated myeloid cells potentially enhances the angiogenic factor VEGF expression and ROS production, CRC cell viability and metabolism. All these Bv8-induced effects may be associated with the activation of Erk signalling pathway during the pro-malignancy process. Bv8 and Bv8-recruited myeloid cells were likely to promote CRC malignancy eventually (**Figure 7**).

The co-localization of myeloid cells and Bv8 in CRC tissue indicated that myeloid cells might be the major source of Bv8 as in line with previous studies. A previous *in vivo* study revealed that Bv8 mRNA was mainly produced in murine bone marrow and myeloid cells (18). In addition, Bv8 expression was up-regulated in tumor-infiltrated CD11b+ Gr1+ cells (19). On the other hand, co-localization of Bv8 and myeloid cells might be caused by chemo-attractive feature of Bv8 (20–22). In this case, a low level of Bv8 would be produced by CRC tumor cells before myeloid cell infiltration and it might recruit myeloid cells towards CRC tumor tissue. Another study argued that Bv8 is likely to be derived from tumor cells (21). Moreover, apart from peripheral leukocytes, Bv8 mRNA was highly expressed in human testis tissue and placenta that may be another source of Bv8 (23).

At the pre-cancer stage of CRC, it is possible to have some myeloid cells around healthy colorectal tissue or inflamed tissue and a low level of Bv8 is secreted from these myeloid cells (24–26). With the migration of myeloid cells, myeloid cell infiltration occurs and Bv8 is accumulated at the same location as infiltrated myeloid cells. Such Bv8-meidates paracrine system may enhance the interaction between immune cells and cancer cells (27). Due to the chemo-attractive feature of Bv8 at a relatively low concentration, Bv8 might be a potential therapeutic target for early CRC management. Bv8 can also serve as a potential targetable chemokine in CRC immunotherapy (28).

We noticed that 1nM of Bv8 induced maximum malignant effects and VEGF expression on Caco-2 cells, but a higher level of Bv8 was ineffective on these. Similarly, another potent neutrophil chemoattractant fMLP showed the maximum recruiting ability with low concentration, but fewer neutrophils were recruited if fMLP concentration increased (29). It is highly likely that the concentration of Bv8 is the key to its tumor-related function in our model. The effect of various Bv8 concentration on myeloid cell-infiltrated CRC model can be potentially addressed in future studies. There might be a possibility that the myeloid-CRC system actively regulates Bv8 at a low level to establish a pro-tumoral microenvironment (for example, myeloid cells might contribute to the negative regulation of Bv8 production in Caco-2 cells, maintaining Bv8 at a relatively low level.), but again, this requires further investigation.

The flow cytometry data demonstrated that CRC cells were more susceptible to co-culture treatment compared to myeloid cells, in terms of ROS production. This suggested CRC cells to be the major source of ROS in a myeloid cell-infiltrated TME of CRC. To support this, our data also showed enhanced ROS production from CRC cells after Bv8 treatment. Combined with our cell viability assay, the enhanced ROS production in CRC cells in a myeloid cell-infiltrated TME might promote cell viability of CRC cells. ROS-induced effect was also reported in studies of polyphenols in CRC, which on the other hand, presenting a cytotoxic feature to CRC cells (30). It is important to consider that different ROS levels led to normal cell homeostasis, tumor-promoting events and tumor-inhibiting events (31). ROS is produced during normal cellular events where antioxidants are also generated to maintain ROS to certain level. Increased ROS causes cell cycle progression, cell proliferation and survival, genomic instability and angiogenesis whilst excessive ROS leads to cell cycle arrest and tumor cell death (31). Our findings again indicated the multi-faced role of Bv8 in CRC microenvironment. It is likely that the concentration of Bv8 is the key of its tumor-related function in our study. Indeed, we noticed that 1 nM Bv8 was optimal to induce ROS and VEGF production in CRC cells. We also found that Bv8 was capable to trigger chemotaxis of neutrophils at a relatively low concentration, which is an essential process for neutrophil infiltration (**Supplemental Figure 2**).

ROS is generated during normal physiological cellular activities, mainly produced in mitochondria and peroxisomes (32). We noticed that Bv8 might up-regulate both ROS production and mitochondrial ATP production in Caco-2 cells. Nevertheless, it is hard to conclude whether ROS and mitochondrial metabolism were increased by Bv8 individually or increased in a reciprocal manner. In mitochondria, glucose uptake and ROS production form an interactive system that involves mitochondrial ROS production, antioxidant process and mitochondrial function (33). A moderate level of ROS enhances glucose uptake, transcription, and expression of glucose transporters (GLUTs), translocation of GLUT1 to plasma membrane and GLUT1 activity in mitochondria (33–35). On the other hand, an increased mitochondrial metabolism also promotes ROS production through higher glycolytic flux until ROS reaches cytotoxic threshold (36). To prevent cell damage, mitochondria initiate antioxidant protection mediated by manganese-dependent superoxide dismutase, dehydroascorbic acid, and pentose phosphate pathway flux (33, 37, 38). While the excessive level of ROS in mitochondria causes vicious co-stimulation between glucose uptake and ROS production, which have been reported in various diseases including cancer (39). Our study introduced Bv8 as another variable into this mitochondrial-metabolism-ROS-production system. Further studies on deeper insights of whether Bv8 affects individual factors in this system or affects the system as a whole are required. It is also worth taking the following arguments into consideration: firstly, our data presented that myeloid cell infiltration also increased ROS production in CRC cells. Given that myeloid cells tend to regulate Bv8 at an optimal level to maximize pro-tumoral effects in our study, the delicate balance of myeloid cell-Bv8 system and Bv8-ROS-mitocondrial-metabolism system should be studied in the future; Moreover, our data demonstrated that Bv8 increased ROS in CRC cells but decreased ROS in myeloid cells. Whether this indicates Bv8-stimulated ROS production is cell type specific or more complicated correlation needs to be investigated further.

The reciprocal effect of cell viability between co-cultured Caco-2 cells (increased) and macrophage-like U937 cells (decreased) may suggest the occurrence of immunosuppression of immune cells, particularly macrophages. It may also indicate increased malignancy of cancer cells once myeloid cells infiltrated into CRC. In agreement with our data, a previous study demonstrated that crosstalk between myeloid-derived suppressor cells and macrophages within CRC microenvironment reduced macrophage activities and production of antitumor cytokine IL-12 (40). Moreover, tumor cell-induced cytokines such as HMGB1 triggered macrophage apoptosis while encouraged tumor cell growth (41). In our study, addition of PKRA7 (Bv8 receptor antagonist) +Bv8 promoted cell viability of Caao-2 cells may be due to that lowering effective level of Bv8 would lead to pro-tumoral effects on CRC cells. Yet, 1 nM of Bv8 seemed to be an optimal concentration in our other experiments *per se*.

Since the population of myeloid cells is heterogenous in CRC, single cell RNA-seq might be worth conducting to categorize which sub-group of myeloid cells is the main source of Bv8 (42, 43). Even though we compared tumor tissue and NAT from the same patients to minimize variable factors, the holistic immune signature of NAT was different from tissue from healthy volunteer or patients with benign tumor (as immune phenotypes are affected by the presence of tumors) (44). Therefore, comparing Bv8 level in tissues from patients with CRC and without CRC (benign tumor for example) would facilitate our understanding of the role of Bv8 in physiological and pathophysiological disease settings. Immunofluorescent staining is well established (45, 46) for the study type reported here but it is semi-quantitative, a quantitative assessment (e.g. western blotting) should be considered in future study. A second myeloid marker such as CD11b and Bv8 mRNA in the *ex vivo* samples are also worth studying. In addition, we used colorectal cell line for current *in vitro* study since they share some level of similarity to CRC cells and is cost effective and easy to use. However, there is limitation in the use of cell lines. According to a pan-cancer survey, cell lines in general only express 2% tissue-specific or 5% tumor-specific genes (47, 48). In order to further investigate the expression of Bv8 in Caco-2, HL60 and U937 cells, PKR2 (Bv8 receptor) silencing or knockout model can be performed. Further study using *in vivo* mice model e.g. cross breeding Cre-Bv8 mice (conditional Bv8 deletion) with APC Min/+ mice (colon cancer) to have Cre-Bv8-APC min/+ mice (49), would be worth approaching. Migration dose response-curve in response to varies concentrations of Bv8 should also be considered for future transwell assay or chemotaxis assay.

In conclusion, despite the limitations described above, this study deepens our understanding of the vital roles of Bv8 in myeloid cell infiltration and CRC development including ROS and ATP generation. This suggest that Bv8 may serve as a potential targeted chemokine in CRC immunotherapy. Due to the chemo-attractive feature of Bv8 at a relatively low concentration, Bv8 might also be a potential therapeutic target for early CRC management.

## Data availability statement

The original contributions presented in the study are included in the article/**Supplementary Material**. Further inquiries can be directed to the corresponding authors.

## Ethics statement

The studies involving human participants were reviewed and approved by Henan Provincial Hospital, Zhengzhou, Henan, China and NRES Committee London - Camden and Islington. Written informed consent for participation was not required for this study in accordance with the national legislation and the institutional requirements.

## Author contributions

XL: contribution to experimental design, data collection, interpretation of results and writing of paper. GB, EC, JZ, KO’D, and NT: contribution to reagent and sample support. JC, HZ, and CH: study support and writing of paper. DM and LY: Conceived the study design, supervised the project, drafted and corrected through the manuscript. All authors contributed to the article and approved the submitted version.
