# Supplementary material for: Bv8 mediates myeloid cell migration and enhances malignancy of colorectal cancer
Source: Front Immunol. 2023 Apr 5;14:1158045. doi: 10.3389/fimmu.2023.1158045 (PMC10113555; doi:10.3389/fimmu.2023.1158045)
Supplement: Supplementary file 2 [file DataSheet_1.docx]

Supplementary Material

Supplemental file 1

**Bv8 mediates myeloid cell migration and enhances malignancy of colorectal cancer**

**Xiaomeng Li^1^, Enqiang Chang^1,2^, Jiang Cui^1^, Hailin Zhao^1^, Cong Hu^1^, Jiaqiang Zhang^2^,** **Kieran P O’Dea ^3^, Nikhil Tirlapur^3^, Gianfranco Balboni^4^, Liming Ying^5*^, Daqing Ma^1*^**

**^*^Correspondence**:

Liming Ying

l.ying@imperial.ac.uk

Daqing Ma

[d.ma@imperial.ac.uk](mailto:d.ma@imperial.ac.uk)


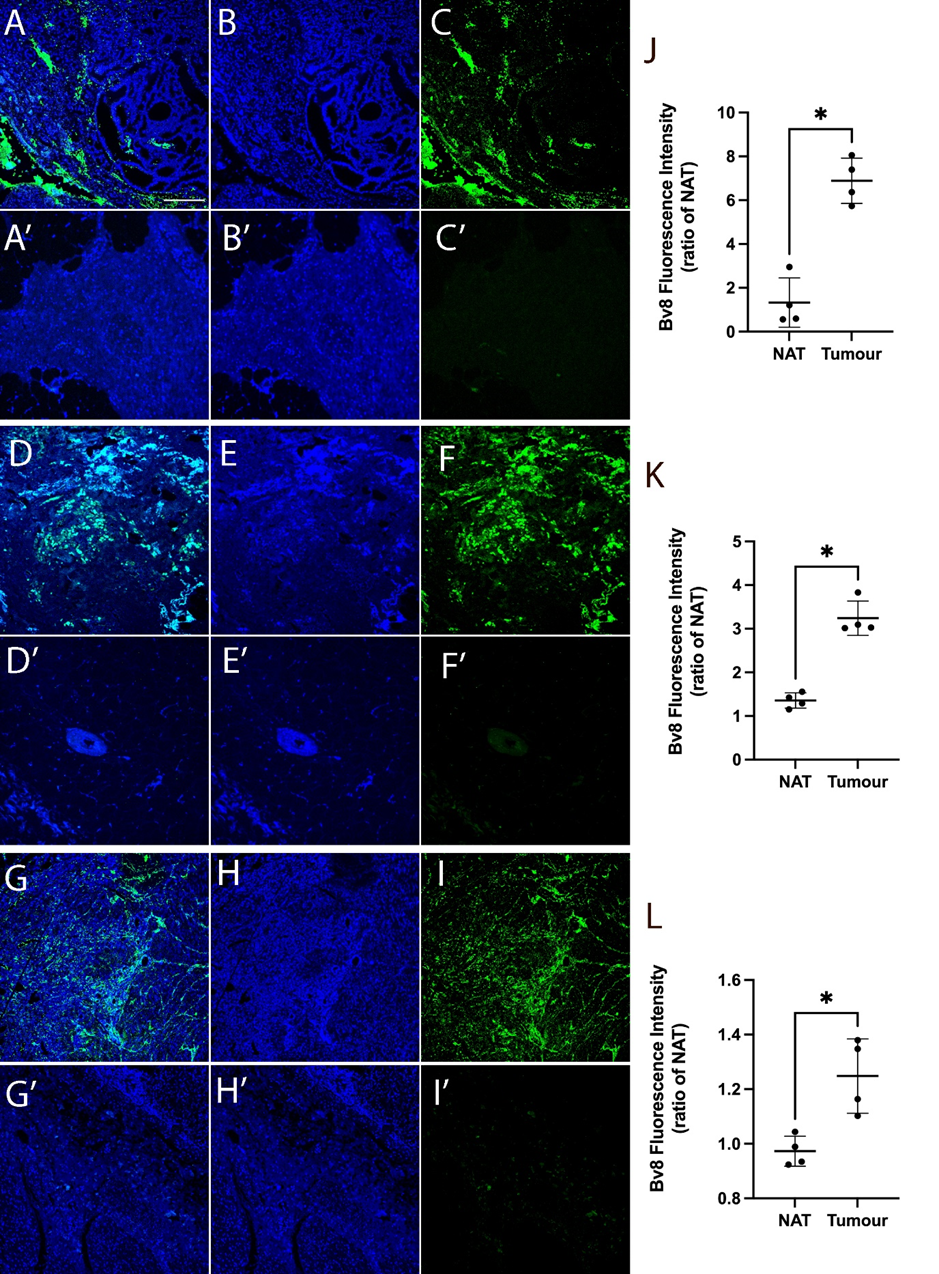


**Supplemental Figure 1. Bv8 expression in tumour tissue and NAT from patients with prostate, breast and colorectal cancer**

Each group of samples were collected from tumour site and respective NAT from the same patient. Samples from patients with prostate cancer (**A-C’**), breast cancer (**D-F’**) and CRC (**G-I’**) were labelled with DAPI (blue) and anti-Bv8 (green) through immunofluorescent staining. Scale bar: 200 μm. Mean fluorescence intensity (ratio of NAT) of Bv8 suggested significantly higher expression in tumour tissue compared to NAT in all three cancer types (**J-L**). Data are presented as dot plot and mean ± SD (n=4). Mann-Whitney U test followed by Dunn’s test was used for statistical analysis. *p<0.05. n refers to the number of different samples from the individual patients.


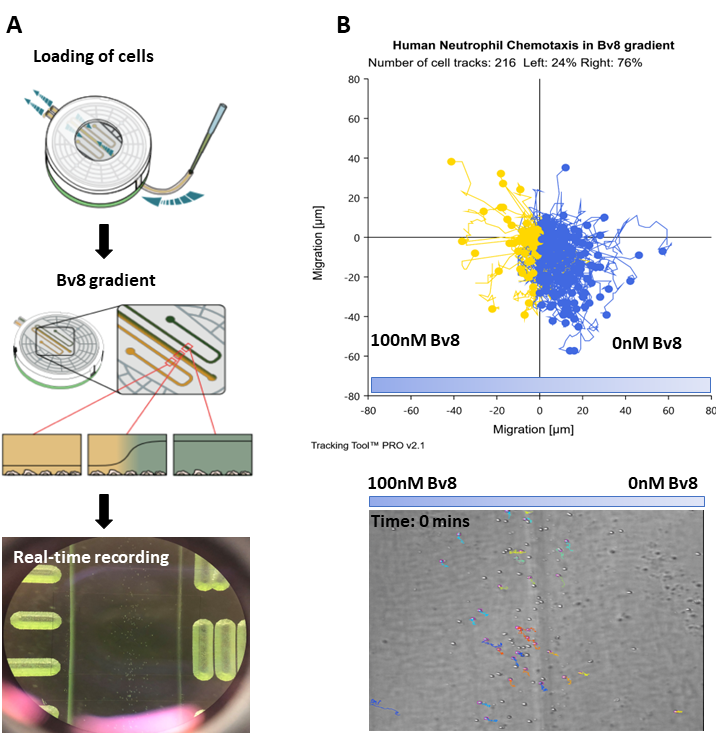


**Supplemental Figure 2. Chemotactic effects of Bv8 on human neutrophils**

**(A)** A real-time chemotaxis system was set up as illustrated in Figure 3C, where 200 µl neutrophils (2x10^6^ cells/ml) were injected into CellDirector 2D. Then 1 ml of chemoattractant (100 ng/ml of Bv8 in RPMI) was pumped into the chamber through a syringe at the speed of 1 µl/min, while the other syringe was pumped with RPMI medium as a control. This led to the formation of a concentration gradient of Bv8 from 0 nM to 100 nM. CellDirector 2D was assembled on a holder under bright field microscopy (10× objective), and bright-field images were collected at 0.5 fpm (1 image every 2 minutes). **(B)** The movement of neutrophils was tracked and analysed using Tracking Tool^TM^ PRO software (top). The bottom figure shows the location of all neutrophils at 0 minute and their moving pattern. Bv8 gradient was set up from 0 nM (right) to 100 nM (left). Real-time video can be found in Supplementary Information.

**
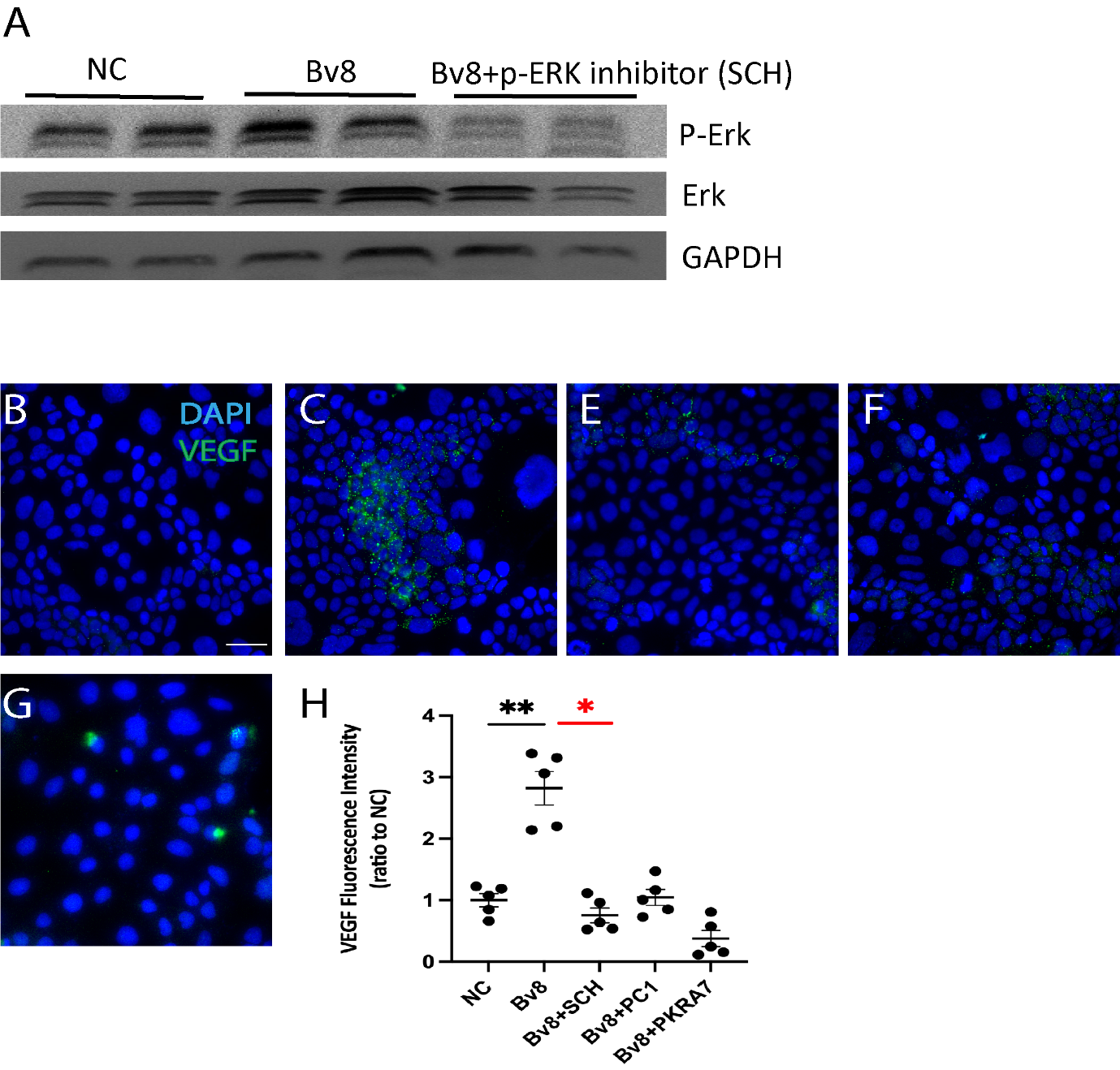
 Supplemental Figure 3.** **Inhibition of Erk signalling pathway supressed Bv8-induced VEGF expression in Caco-2 cells**

**(A)** After evaluating that Bv8 might contribute to the activation Erk signalling pathway, we then explored the role of Erk in Bv8-induced VEGF expression. Successful inhibition of Erk activation was achieved by addition of SCH772984 as shown in the western blotting. **(B-G)** Caco-2 cells were treated with no treatment (NC), 1 nM Bv8, 500 nM SCH in DMSO + 1 nM Bv8, 1 µM PC-1 + 1 nM Bv8 and 1 μg/ml PKRA7 + 1 nM Bv8 for 24 hours. VEGF expressions were assessed by immunofluorescent staining. **(H)** VEGF fluorescence intensity was presented as ratio to naïve control. All data are presented as mean ± SD (n = 5; the number of independent experiments). Student’s t test was used for statistical analysis. *p<0.05, **p<0.01. NC: naïve control.

**
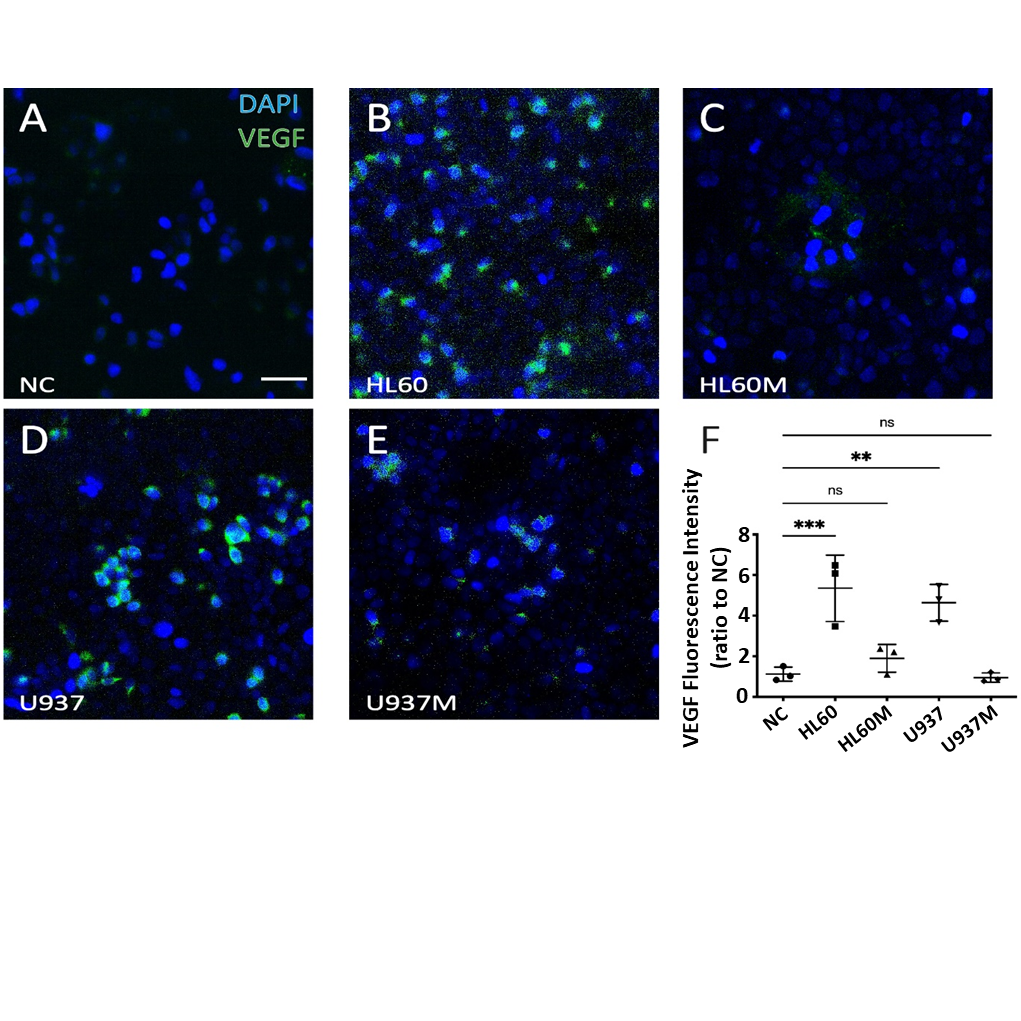
**

**Supplemental Figure 4. VEGF expression in co-cultured CRC cell line**

In order to study the effect of myeloid cell infiltration on VEGF production within TME, Caco-2 cells were co-cultured with either myeloid cells or medium extracted from myeloid cell culture. **(A)** Immunostaining of VEGF expression (green) in untreated Caco-2 cells (naïve control). **(B)** VEGF expression in Caco-2 cells treated with HL60. **(C)** VEGF expression in Caco-2 cells treated with medium extracted from HL60. **(D)** VEGF expression in Caco-2 cells treated with U937. **(E)** VEGF expression in Caco-2 cells treated with medium extracted from U937. **(F)** The ratio of the fluorescence intensity of VEGF compared naïve control. All data were presented as mean ± SD (n =3; the number of independent experiments). Mann-Whitney U test followed by Dunn’s test was used for statistical analysis. **p<0.01, ***p<0.001, ns: not significant. Scale bar: 50 μm, NC: naïve control, HL60: co-cultured with HL60 cells, HL60M: co-cultured with HL60 medium, U937: co-cultured with U937 cells, U937M: co-cultured with U937M.


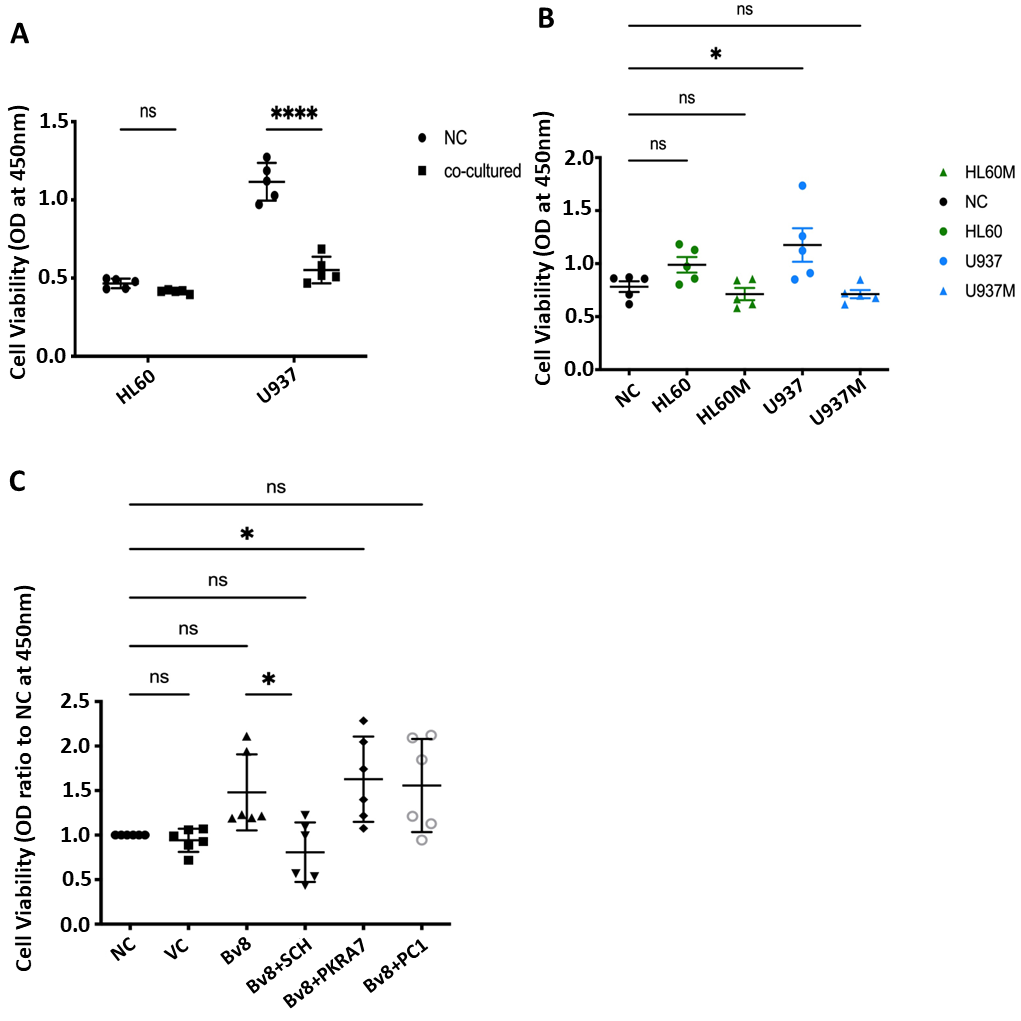


**Supplemental Figure 5. Cell viability of Caco-2 cells and myeloid cells after co-culture or Bv8 treatment**

**(A)** In the co-culture experiment, myeloid cell lines (HL60 cells and U937 cells) were treated with Caco-2 cells for 24 hours (n=4) and viability of U937 cells showed significant increase after treatment. **(B)** Caco-2 cells were treated with HL60 cells, U937 cells and medium extracted from both cell culture for 24 hours (n=5). Among all the treatment, only co-culturing with U937 cells significantly increased cell viability of Caco-2 cells. **(C)** 1 nM Bv8, 500 nM SCH in DMSO + 1 nM Bv8, 1 μM PC-1 + 1 nM Bv8, 1 μg/ml PKRA7 + 1 nM Bv8 and DMSO (vehicle control) were given to Caco-2 cells for 24 hours. No significant change of cell viability was observed after addition of Bv8. All data were presented as mean ± SD (n =5; the number of independent experiments). One-way ANOVA was used for statistical analysis followed by Student-Newman-Keuls test. *p<0.05, ****p<0.0001, ns: not significant. NC: naïve control, HL60: co-cultured with HL60 cells, HL60M: co-cultured with HL60 medium, U937: co-cultured with U937 cells, U937M: co-cultured with U937M, VC: vehicle control.


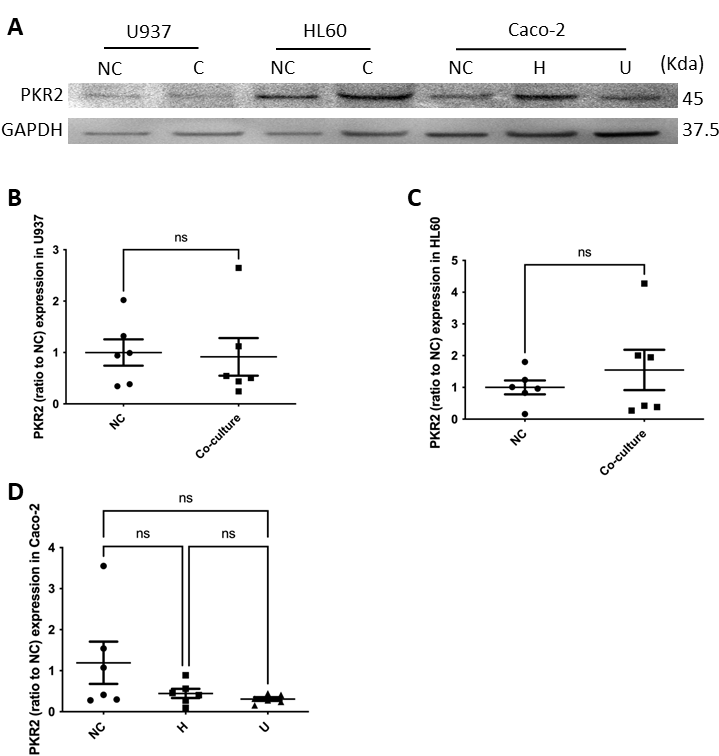


**Supplemental Figure 6. PKR2 expression on myeloid cells and CRC cells after co-culture**

**(A)** After 24-hour co-culture of U937 or HL60 cells with Caco-2 cells, protein expression of PKR2 on each cell type was assessed by western blotting. **(B)** The densitometry of PKR2 was presented as ratio to naïve control in U937 cells. **(C)** The densitometry of PKR2 was presented as ratio to naïve control in HL60 cells. **(D)** The densitometry of PKR2 was presented as ratio to naïve control in Caco-2 cells. (n=6; the number of independent experiments). All data were presented as mean ± SD. Student’s t test was used for statistical analysis. *p<0.05, ns: not significant. NC: naïve control, C: co-cultured with Caco-2 cells, H: co-cultured with HL60 cells, U: co-cultured with U937 cells.
